# The Effect of Chinese Traditional Exercise-Baduanjin on Physical and Psychological Well-Being of College Students: A Randomized Controlled Trial

**DOI:** 10.1371/journal.pone.0130544

**Published:** 2015-07-09

**Authors:** Moyi Li, Qianying Fang, Junzhe Li, Xin Zheng, Jing Tao, Xinghui Yan, Qiu Lin, Xiulu Lan, Bai Chen, Guohua Zheng, Lidian Chen

**Affiliations:** 1 College of Rehabilitation Medicine, Fujian University of Traditional Chinese Medicine, Fuzhou, Fujian, China; 2 Department of Physical Education, Fujian University of Traditional Chinese Medicine, Fuzhou, Fujian, China; 3 Fujian University of Traditional Chinese Medicine, Fuzhou, Fujian, China; Vanderbilt University, UNITED STATES

## Abstract

**Background:**

The physical and mental health of college students tends to continuously decline around the world, therefore, it is important to improve their health during college period. Baduanjin, a traditional Chinese exercise which combines movements with breath and mind, may be one of the selectable effective exercises. However, the effect of Baduanjin exercise on college students has not been established. In this study, we systematically assessed the effectiveness and safety of Baduanjin exercise on physical and mental health of college students by a rigorous randomized, parallel-controlled design.

**Methods:**

A total of 222 college students from Fujian University of Traditional Chinese Medicine were recruited and randomly allocated at an equal ratio into control or Baduanjin training. Participants in control group were informed to maintain their original activity habit, and those in Baduanjin exercise group received a 12-week Baduanjin exercise training with a frequency of 1 hour per day and 5 days per week on the basis of their original activity habit. The physical and psychological outcomes, including lumbar muscle strength, lower limb proprioception function, physical fitness, as well as self-reported symptom intensity, stress, self-esteem, mood, quality of life, quality of sleep, and adverse events, were evaluated at baseline, 13 weeks (at the end of 12-week intervention), and 25 weeks (after the 12-week follow-up period). Intention-to-treat analysis was performed for the above outcomes.

**Results:**

Compared with controls, significant improvements in Baduanjin exercise group at the end of 12-week intervention period were found on lower limb proprioception function (the rate of average trace error on right lower limb (%): control 23.50±5.50, Baduanjin 21.92±6.54, *P*=0.004; the rate of average trace error on left lower limb (%): control 22.32±6.62, Baduanjin 20.63±4.62, *P*=0.046), cardiorespiratory endurance (step test index: control 47.66±5.94, Baduanjin 50.07±9.30, *P*=0.025), flexibility (control 14.35±7.26cm, Baduanjin 15.39±6.43cm, *P*=0.009) and explosive force of lower limb (standing long jump test (m): control 1.77±0.24, Baduanjin 1.79±0.22, *P*=0.005 for adjustment baseline) in physical outcomes, and attention (Schulte Grid test (second): control 210.4±51.15, Baduanjin 192.4±47.14, *P*=0.034) in mental outcome. Lumbar muscle strength in Baduanjin group had been moderately enhanced but no significant difference compared to controls. No significant changes in other physical and mental outcomes, including vital capacity, blood pressure, heart rate, hand grip force, self-symptom intensity, stress, self-efficacy, quality of life, and quality of sleep, were found between groups. No adverse event was reported during the study period.

**Conclusion:**

Regular Baduanjin exercise had an advantage for college students on improvement of lower limb proprioception, enhance of cardiorespiratory endurance, flexibility, explosive force of lower limb and attention, compared with usual exercise.

**Trial Registration:**

Chinese Clinical Trial Registry ChiCTR-TRC-13003329 http://www.chictr.org

## Introduction

With the progress of science and technology, the integration of digital technology within daily life is becoming the cultural norm. More young people spend a majority of their spare time in the digital devices [1, 2]. College students are the vanguard of the first cohort of young people, and sedentary lifestyle becomes prevalent among college students globally [3–5]. A report from American College Health Association indicated that only 43.6% college students reported vigorous physical activity for 20 minutes or moderate activity for 30 minutes on only 3 or fewer days of the previous 7 days [6, 7]. Participation in large amounts of sedentary lifestyle is associated with multiple health problems such as impaired lipid profiles and glucose uptake [8]. As a result, college students may be at increased risk for the metabolic syndrome and susceptible to serious chronic diseases later in life [9–11]. Similarly, mental health problems in college students are increasing steeply due to peer pressure and environmental changes, and becoming a worldwide public health burden [12–14]. In the United States, mental disorders accounted for nearly half of the disease burden for young adults [15]. These mental health problems are associated with poorer academic achievements, instable intimate relationship, even suicide [16]. However, only a small part of college students with mental distress ask for and receive adequate attention [17]. It is well established that physical inactivity in young adults are directly linked to mental distress [18,19]. Therefore, physical activity may play an important role in the management of mild-to-moderate mental distress [20,21].

Regular physical activities or exercises have been demonstrated to benefit participants’ physical, mental, and social well-being [22–24]. Recent studies showed that exercise for 8 to 12 weeks was effective for promoting physical fitness and mental health, and improving body composition in college students [25,26]. Baduanjin exercise, translated as the ‘eight section of brocades’, is one of many traditional Chinese Qigong exercises for health promotion. It includes eight separate, delicate, and smooth exercise movements, and each movement benefits different physical parts of body or particular organs [27]. Baduanjin exercise can promote Qi function through the whole exercise of body posture, movement, breathing, and meditation—that means using the natural energies to optimize and balance internal energy with the coordination of body, breath, and mind [28]. Current studies have suggested that Baduanjin training appeared to have substantive benefits for older adults [29–31]. A few studies for young adults also indicated that Baduanjin exercise provides potential benefits on reducing depression, stress and anxiety, building self-control and healthy mind, and enhancing physical function [32–34]. However, due to the methodological limitations in previous studies, whether Baduanjin exercise can be recommended as an effective exercise to enhance college students’ health is still unknown. The purpose of this trial is to systematically evaluate the effect of Baduanjin exercise on physical and psychological outcomes of the college students in China.

## Methods and Design

### 2.1 Study design

This was a randomized, single-blind, parallel-controlled trial to evaluate the effectiveness and safety of Baduanjin exercise for the physical and mental health of college students. A total of 222 eligible college students from the Fujian University of Traditional Chinese Medicine (FJTCM) were recruited and randomly allocated to either control or Baduanjin exercise group with a ratio of 1:1. The relative physical and psychological outcomes were measured at baseline, 13 weeks (at the end of intervention), and 25 weeks (after the 12-week follow-up period). The design of the study was detailed in the published protocol [35].

### 2.2 Ethics approval

This trial was carried out in accordance with the declaration of Helsinki and approved by the Ethics Board of FJTCM (approval number: 2013, Reviewed-No. 043). All participants provided written informed consent prior to participation. The individual in this manuscript has given written informed consent (as outlined in PLOS consent form) to publish these case details.

### 2.3 Participants

Participants were college students recruited from the first or second grade in FJTCM through the campus poster, advertisement, and campus broadcast between September 9 and September 19, 2013.

Participants were eligible to participate in the study if they met the following criteria: aged 18 to 25 years; provided the informed consent; and were freshmen or sophomores. Participants were excluded if they had been engaged in a long-term regular practice of Baduanjin, or were members of the Martial Arts Association, Dance Association, Aerobics Association, Sanda Association, or Taekwondo Association of FJTCM; or if they had suffered from severe cardiovascular diseases, musculoskeletal system diseases, or sports contraindications. Baseline assessments were conducted within 2 weeks before the beginning of the intervention program.

### 2.4 Randomization, allocation concealment and blinding

The random allocation sequence was produced by an independent statistician who worked in the Evidence-Based Center of FJTCM via the PLAN sentences of the statistical software SAS9.1. The detail of random allocation sequence was kept with a project manager. After the baseline assessments, the project manager informed each eligible participant his/her group (Baduanjin exercise or control group) via cell phone.

Although it was impossible to blind the participants and exercise instructors, we masked participants’ allocation information to outcome assessors, and urged participants not to disclose their allocation information during the period of outcomes measurement. In addition, we built the blind code of allocation to blind the statistician, and in which, the group assignment (Baduanjin exercise or usual exercise control group) was replaced by the alphabet A or B.

### 2.5 Intervention and follow-up

#### 2.5.1 Control group

Participants in the control group were informed to keep their original physical activity habit during the 12-week intervention period.

#### 2.5.2 Baduanjin exercise group

On the basis of their original physical activity habit, participants in the Baduanjin exercise group gathered and practiced 1 hour Baduanjin exercise at 5 p.m. each day with a frequency of 5 day per week at the gymnasiums of the university. Baduanjin exercise was instructed by two qualified instructors who had engaged in the physical education over 5 years. The training scheme of Baduanjin exercise was in accordance with the *Health Qigong—Baduanjin* published by the General Administration of Sport of China, which consisted of 10 postures (including the beginning and ending posture) [36].

Intervention period lasted 12 weeks from September 23, 2013 to December 15, 2013. All participants were required to fill in the daily activity log during the intervention period, in which the type, frequency and duration of participant’s activity in a whole day was recorded and classified into low, moderate, or high intensity grade. An unsupervised 12-week follow-up period for each participant began on December 16, 2013, and ended on March 7, 2014. No participants received additional exercise intervention but were required to fill in the daily activity log during the 12-week follow-up period.

### 2.6 Outcome assessment

The primary measure of outcomes was the change in the lumbar muscle strength, lower limb proprioception function, as well as self-reported symptom intensity, stress, self-efficacy and attention measured at 13 weeks (the first week after completion of the 12-week intervention period). We also evaluated the long term effect over a 12-week period at 25 weeks (the first week after completion of 12-week follow-up period). Lumbar muscle strength (consisting of flexion, and rotation myodynamia) and lower limb proprioception function were assessed using the *Tergumed Work Station* (produced by *Proxomed GmbH*, *Germany*) and the *Prokin* proprioception evaluation and training system (produced by *Tecnobody*.*S*.*r*.*l*, *Italy*.) by the blinded operators at the Evaluation Department of Rehabilitation Hospital Affiliated to FJTCM. The scores of self-reported symptom intensity, stress, self-efficacy and attention were felt to best reflect the wellbeing health condition of college students, and were conducted using their corresponding scales with the confirmed validity [37–39].

Secondary outcomes included changes in physical fitness, mood and mindfulness, self-esteem, quality of life, and quality of sleep measured at 13 weeks and 25 weeks. Physical fitness, consisting of cardiopulmonary function, hand grip force, explosive force of lower limb and flexibility, was measured using the step test, hand grip strength test, standing long jump and the ‘sit and reach’ test respectively, which has been shown to be a reliable and responsive measure in overall physical fitness of college students according to the *Chinese University Students’ Physical Health Standards*. The psychological health and life or sleep quality outcomes were collected via the self-reported questionnaires at the Center of College Student Activity of FJTCM. Details about the outcome assessment were described in the published protocol of this trial [35].

### 2.7 Sample size and statistical analysis

No previous study of Baduanjin exercise for the physical and mental well-being in college students with rigorous design was available. Although Baduanjin exercise as a whole may be broken down into eight separate movement form which each focuses on different physical area, majority of them need rotate the body on waist when practicing [40]. Therefore the change of lumbar muscle strength was established to estimate the sample size. Our preliminary data from 20 college students indicated the means with standard deviation of the flexion strength of lumbar muscle was 258.25 newton (N) and 114.19 N, respectively. The current trial was designed to detect a 20% increase on lumbar flexion muscle strength after the Baduanjin exercise intervention for 12-week period [41,42]. A sample size of 101 per group with a type I error of 5% (α = 0.05) and 90% power (β = 0.10) was necessary. With an estimated dropout rate of 10%, a final sample size of 111 per group was required.

The primary and secondary outcomes were analyzed on an intention-to-treat (ITT) basis. Method of Last Observation Carry Forward was applied to fill in the missing data. Analyses were done using SPSS 21.0 (IBM, Chicago, IL, USA) software packages. The statistical significance was defined as two-sided *P* value of <0.05.

In descriptive analysis of the sample, continuous variables were expressed as mean and standard deviation for normal distribution, and median and inter-quartile range for non-normal distribution. Normality was tested using Kolmogorov-Smirnov test. Appropriate transformations were applied in cases of non-normal distribution. Categorical variables were expressed as proportions with their standard error.

Baseline characteristics between groups were compared using the *t*-test or Mann-Whitney test for continuous variables and Pearson chi-squared or Fisher’s exact test for categorical variables. If incomparability appears, the inequality factors were treated as confounding variables in the final efficacy analysis.

For comparison of the primary or secondary outcomes between groups, a *t*-test or non-parametric tests was used for continuous variables, and Pearson chi-squared or Fisher exact test for categorical variables. To control for possible confounding factors, linear regression model was applied for dependent continuous variables and logistic regression models for dependent categorical variables. Subgroup analysis stratified by gender was conducted for the primary outcomes.

## Results

### 3.1 Baseline characteristics

**Fig 1:**
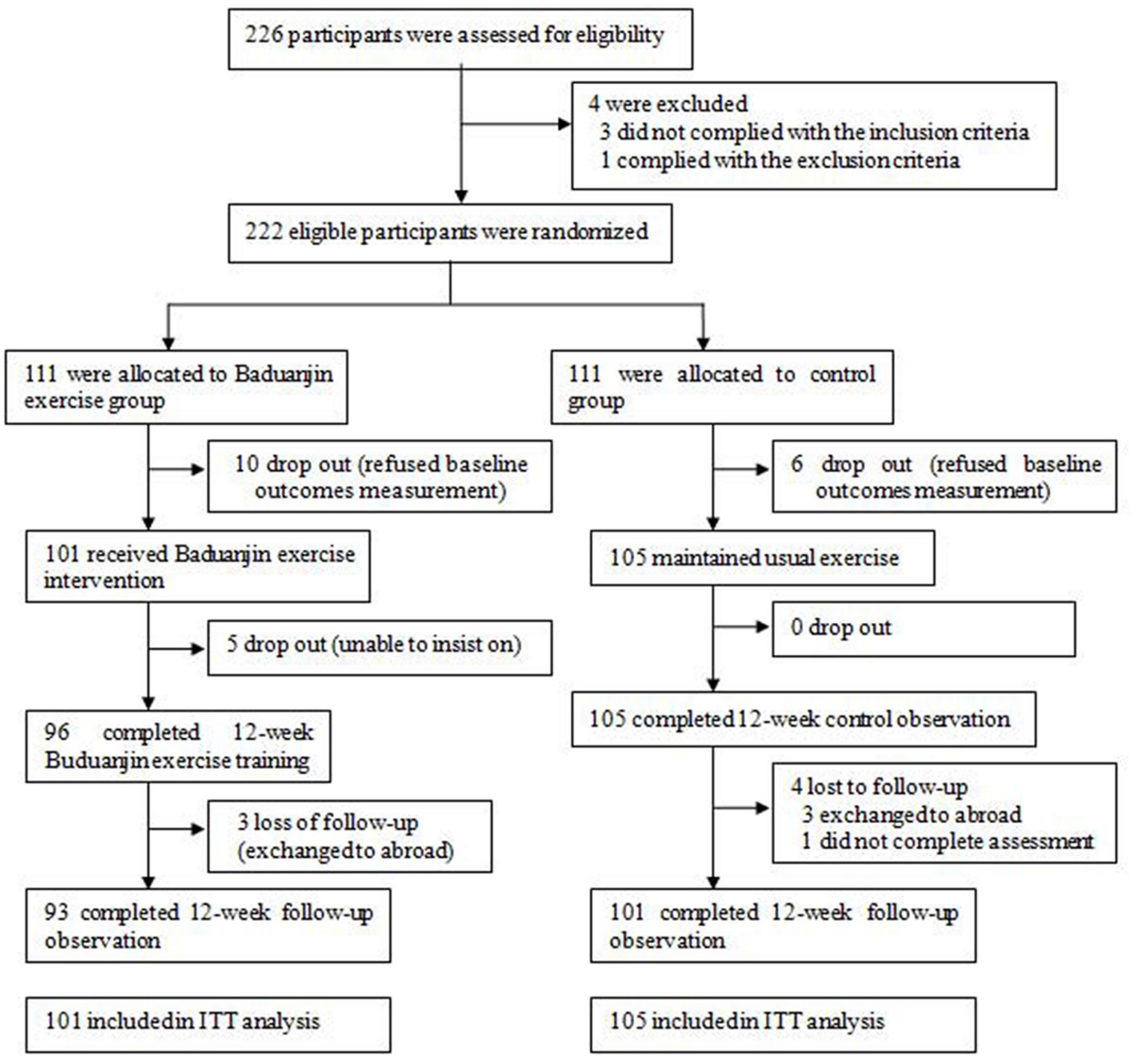
Participant flow through the study reported in a CONSORT diagram.

Fig 1 illustrates the participants flow. 16 participants (10 in Baduanjin exercise group and 6 in control) dropped out before baseline assessment. Therefore they were excluded from the ITT analysis. During the 24-week study period (including 12-week intervention period and 12-week follow-up period), 5 participants dropped out and 7 participants lost to follow-up, with 93 (92.1%) participants in Baduanjin exercise group and 101 (96.2%) participants in control group. There was no statistically significant difference between groups in terms of drop-out or lost to follow-up (χ^**2**^ = 1.586, *P* = 0.208). Of 96 participants in the Baduanjin exercise group with the completed 12-week Baduanjin exercise training, 18 participants had 75% attendance rate (actual training days/plan training days) or less, and 38 participants possessed 76%-85%, 40 participants attained more than 85% attendance rate. Reasons given for not attending training mainly included training time clashing with other commitments and illness.

In 206 eligible participants, 82.5% of them were female students; the average age of participants was 20.78 years old. Baseline demographic characteristics between groups including sex proportion, the average of age, baseline exercise time, height and weight had no significant difference (Table 1). The average time of high intensity activities, low intensity activities, and sedentary time between groups were not statistically different whether in the intervention period or the follow-up period. The average time of moderate intensity activities in Baduanjin exercise group were higher than the control group during 12-week intervention period (*P* = 0.001) (Table 2).

**Table 1:** Baseline characteristics between Baduanjin exercise and control groups.

Characteristics	Baduanjin exercise group (n = 101)	Control group (n = 105)	Total
Total included in the final analysis	101	105	206
Sex: Females (%)	86 (85.1)	84 (80.0)	170 (82.5)
Age–yr (Mean±SD)	20.63±1.03	20.92±1.15	20.78±1.10
Height-cm (Mean±SD)	160.86±6.87	160.21±6.70	160.53±6.78
Weight-kg (Mean±SD)	53.02±7.41	53.08±8.03	53.05±7.71
Baseline exercise time*–min	34.08±20.92	37.53±27.81	35.81±23.21

*Baseline exercise time was the self-reported mean time of undergone moderate-vigorous exercises in the last one month.

**Table 2:** Comparison of average activities time during the 12-week intervention and 12-week follow-up period between groups (hours)

Characteristics	Baduanjin exercise group (n = 101)	Control group (n = 105)	*P* value
Average activities time during the intervention period (1–12 week)*			
Sedentary time	7.77±2.83	7.71±3.16	0.881
low intensity activities time	1.81±0.70	1.97±0.98	0.183
moderate intensity activities time	1.06±0.35	0.62±0.37	0.001
high intensity activities time	0.14±0.21	0.17±0.20	0.289
Average activities time during the follow-up period (13–24 week) *			
Sedentary time	6.88±3.15	6.85±3.29	0.945
low intensity activities time	2.23±1.30	2.18±1.46	0.793
moderate intensity activities time	1.09±1.18	0.90±0.89	0.196
high intensity activities time	0.19±0.58	0.24±0.63	0.617

* *Sedentary time* were defined as the time of sitting down with waking state, such as attending classes, reading, playing computer, etc. *Low intensity activities* were defined as those with energy consumption less than 3.0 Metabolic Equivalents (METs) including walking, general housework, mild stretching exercise, etc. *Moderate intensity activities* were defined as those with energy consumption range from 3.0 to 6.0 METs including Baduanjin exercise, brisk walking, jogging, cycling, stretching exercise, etc. *High intensity activities* were defined as those with energy consumption over 6.0 METs including running, playing ball games, mountaineering, etc.

### 3.2 The effect of Banduanjin exercise on primary physical and psychological outcomes

Although participants in Buduanjin exercise group had a mean improvement at the end of 12-week exercise of 42.24N, 52.13N, and 38.67N, respectively on flexion, left rotation, and right rotation, the difference was not statistically significant when compared with control group. The lower limb proprioception function in participants of Baduanjin exercise group had an obvious improvement after 12-week regular exercise. The rate of average trace error (ATE) on right, and left lower limb decreased a mean of 7.29, 10.16 percent, respectively, while controls had a mean decline of 6.76, 9.59 percent respectively, for a significantly statistical difference (*P* = 0.004, *P* = 0.046, respectively). The primary psychological outcomes on well-being included self-reported psychological symptom intensity, stress, self-efficacy, and attention, which were assessed by using their corresponding questionnaires with the definite validity and reliability. No significant change in self-reported psychological symptom intensity, stress, self-efficacy was observed at the end of 12-week intervention period, respectively. However, the improvement of attention was significant, and participants with Baduanjin exercise improved a mean of 21.51 second over 12 weeks, while control group participants had a mean improvement of 14.24 second over 12 weeks after adjusting for the baseline values (*P* = 0.034) (Table 3). The above primary outcomes after additional 12-week follow-up were not different between groups (Table 3).

**Table 3:** Primary physical and psychological effects.

Outcomes	Groups	Numbers of participants	Baseline	P value	End of 12 weeks intervention period	Difference of 12 week intervention to baseline	*P* ^§^ value	End of 12 week follow-up period	Difference of 12 week follow-up to baseline	*P* ^§^ value
			Mean (SD)		Mean (SD)	Mean (SD)		Mean (SD)	Mean (SD)	
Lumbar muscle strength										
Flexion (N)^#^	BDJ	101	254.58 (109.32)		296.82 (112.64)	42.23 (71.41)		299.8 (106.6)	45.18 (63.38)	
	Con	105	252.44 (125.49)	0.896	304.21 (135.82)	51.77 (64.47)	0.728	298.9 (125.6)	46.45 (68.32)	0.641
Rotation left (N)^#^	BDJ	101	218.70 (120.59)		270.83 (151.18)	52.12 (86.92)		276.9 (130.2)	58.15 (77.58)	
	Con	105	239.12 (159.39)	0.993	289.05 (167.77)	49.93 (89.38)	0.934	290.2 (174.2)	51.09 (94.21)	0.668
Rotation right (N)^#^	BDJ	101	226.37 (113.66)		265.04 (125.93)	38.67 (67.37)		278.0 (125.0)	51.64 (87.10)	
	Con	105	237.07 (150.92)	0.638	268.59 (150.85)	31.52 (86.88)	0.588	277.7 (157.7)	40.60 (88.90)	0.432
Lower limb proprioception function										
Right lower limb ATE (%)^#^	BDJ	101	29.21 (12.75)		21.92 (6.54)	-7.30 (12.51)		37.81 (8.81)	8.60 (16.31)	
	Con	105	30.26 (13.91)	0.574	23.50 (5.50)	-6.76 (12.10)	**0.004**	39.14 (10.22)	8.89 (18.49)	0.459
Left lower limb ATE (%)	BDJ	101	30.79 (13.06)		20.63 (4.62)	-10.16 (12.07)		36.41 (8.24)	5.62 (16.43)	
	Con	105	31.91 (17.04)	0.319	22.32 (6.62)	-9.59 (15.96)	**0.046**	36.29 (8.69)	3.38 (21.15)	0.764
Psychological outcomes										
Self-symptom intensity (SCL-90 scale, score)	BDJ	101	142.9 (33.58)		135.6 (31.36)	-7.30 (22.42)		130.6 (34.83)	-12.33 (27.43)	
	Con	105	142.1 (32.77)	0.865	136.2 (32.43)	-5.96 (24.58)	0.713	130.4 (31.94)	-11.70 (29.68)	0.923
Stress, score (CPSS scale)	BDJ	101	24.22 (5.18)		23.53 (5.40)	-0.69 (4.67)		22.72 (5.72)	-1.50 (5.16)	
	Con	105	23.91 (5.50)	0.684	22.60 (5.43)	-1.31 (4.38)	0.207	23.22 (5.72)	-0.70 (6.11)	0.352
Self-efficacy, score (GSES score)	BDJ	101	2.46 (0.44)		2.49 (0.43)	0.03 (0.44)		2.50 (0.45)	0.04 (0.47)	
	Con	105	2.59 (0.40)	0.036	2.63 (0.46)	0.04 (0.33)	0.242	2.61 (0.46)	0.02 (0.40)	0.503
Attention, second (Schulte Grid test)	BDJ	101	213.9 (58.84)		192.4 (47.14)	-21.51 (55.7)		193.9 (54.31)	-20.04 (65.86)	
	Con	105	224.6 (47.52)	0.151	210.4 (54.15)	-14.24 (60.7)	**0.034**	202.8 (58.34)	-21.81 (69.14)	0.422

^#^ performed by non-parameter test;

^§^adjusted for baseline

### 3.3 The effect of Baduanjin exercise on secondary physical and psychological outcomes

On the physical parameter for the cardiorespiratory endurance was measured by using a method of step test, in which higher test index indicated better cardiorespiratory endurance. Compared to their baseline data, participants in control group had a decline of 5.62 point as well as a decline of 1.4 point in the Baduanjin exercise group at the end of 12-week intervention period with a significant difference between groups (*P* = 0.025). Participants in Baduanjin exercise group had obvious improvement on physical parameters including flexibility, explosive force of lower limb compared with the controls at the end of 12-week intervention period (*P* = 0.009, *P* = 0.005, respectively) after adjusting for baseline values (Table 4). Other physical parameters, such as vital capacity, blood pressure, heart rate, and hand grip force, had no statistical difference at the end of intervention period between groups (Table 4). No significant change was observed at the end of 12-week intervention period or after 12-week follow-up period between groups for the other secondary psychological outcomes included self-esteem, mood and mindfulness, quality of life, and quality of sleep (Table 4).

**Table 4:** Secondary physical and psychological effects.

Outcomes	groups	numbers	Baseline	*P* value	End of 12 weeks intervention period	Difference of 12 week intervention to baseline	*P* ^*§*^ value	End of 12 week follow-up period	Difference of 12 week follow-up to baseline	*P* ^*§*^ value
			Mean (SD)		Mean (SD)	Mean (SD)		Mean (SD)	Mean (SD)	
Physical fitness										
Cardiorespiratory endurance (Step test index)	BDJ	101	51.47 (13.87)		50.07 (9.30)	-1.40 (16.42)		51.95 (10.54)	0.48 (15.90)	
	Con	105	53.28 (12.68)	0.328	47.66 (5.94)	-5.62 (13.69)	**0.025**	50.60 (10.59)	-2.69 (13.27)	0.249
Vital capacity, ml	BDJ	101	2636.5 (495.73)		2406.2 (676.26)	-230.3 (438.90)		2495.2 (603.2)	-141.3 (406.8)	
	Con	105	2687.7 (574.50)	0.495	2531.3 (630.93)	-156.4 (356.45)	0.226	2591.2 (629.5)	-96.54 (481.4)	0.473
SBP, mmHg	BDJ	101	117.28 (13.04)		112.01 (15.91)	-5.27 (14.59)		109.7 (10.88)	-7.59 (10.35)	
	Con	105	115.04 (12.27)	0.287	111.16 (10.41)	-4.24 (10.63)	0.861	108.7 (11.91)	-6.71 (11.60)	0.508
DBP, mmHg	BDJ	101	69.52 (8.62)		66.57 (8.18)	-2.96 (8.37)		65.58 (7.91)	-3.95 (8.53)	
	Con	105	69.74 (10.49)	0.870	66.93 (11.42)	-2.81 (14.13)	0.862	64.95 (7.20)	-4.80 (9.12)	0.358
Heart rate, bpm	BDJ	101	83.61 (13.06)		82.61 (14.33)	-1.00 (14.74)		82.69 (16.99)	-0.93 (18.52)	
	Con	105	80.49 (13.04)	0.087	82.22 (12.27)	1.73 (13.13)	0.407	85.00 (14.87)	4.51 (13.86)	0.053
Flexibility, cm	BDJ	101	13.17 (6.67)		15.39 (6.43)	2.22 (3.26)		13.05 (6.24)	-0.12 (3.65)	
	Con	105	13.32 (7.46)	0.882	14.35 (7.26)	1.04 (3.34)	**0.009**	12.26 (7.35)	-1.06 (4.46)	0.116
Standing long jump, m	BDJ	101	1.74 (0.23)		1.79 (0.22)	0.056 (0.12)		1.75 (0.22)	0.016 (0.13)	
	Con	105	1.77 (0.25)	0.362	1.77 (0.24)	-0.006 (0.14)	**0.005**	1.76 (0.26)	-0.009 (0.14)	0.175
Hand grip force, N	BDJ	101	28.89 (8.46)		29.10 (7.22)	0.22 (5.39)			NS	
	Con	105	29.37 (8.07)	0.677	29.11 (7.16)	-0.25 (6.20)	0.990		NS	
Self-esteem, score (SES scale)	BDJ	101	31.17 (3.69)		31.56 (3.30)	0.39 (3.35)		30.81 (3.45)	-0.36 (3.54)	
	Con	105	31.41 (3.29)	0.621	31.31 (3.27)	-0.10 (2.47)	0.278	31.00 (3.71)	-0.41 (3.15)	0.904
Mood and mindfulness, score (POMS scale)	BDJ	101	102.3 (16.14)		106.0 (15.68)	3.63 (14.09)		103.8 (16.78)	1.42 (18.69)	
	Con	105	103.5 (17.34)	0.631	107.4 (17.95)	3.92 (18.04))	0.682	104.6 (16.89)	1.16 (18.09)	0.849
Quality of life, score (WHOQOL-BREF scale)	BDJ	101	55.84 (6.65)		55.09 (6.93)	-0.76 (5.90)		56.29 (7.45)	0.44 (6.43)	
	Con	105	54.94 (6.45)	0.323	54.26 (7.02)	-0.68 (5.27)	0.798	55.61 (7.45)	0.67 (6.04)	0.896
Quality of sleep, score (PSQI scale)	BDJ	101	3.67 (1.48)		4.77 (1.78)	1.09 (1.87)		3.56 (1.62)	-0.11 (1.81)	
	Con	105	4.08 (1.73)	0.073	5.12 (1.66)	1.05 (1.64)	0.443	3.79 (1.80)	-0.29 (1.80)	0.810

^§^adjusted for baseline

### 3.4 Subgroup analysis

**Fig 2:**
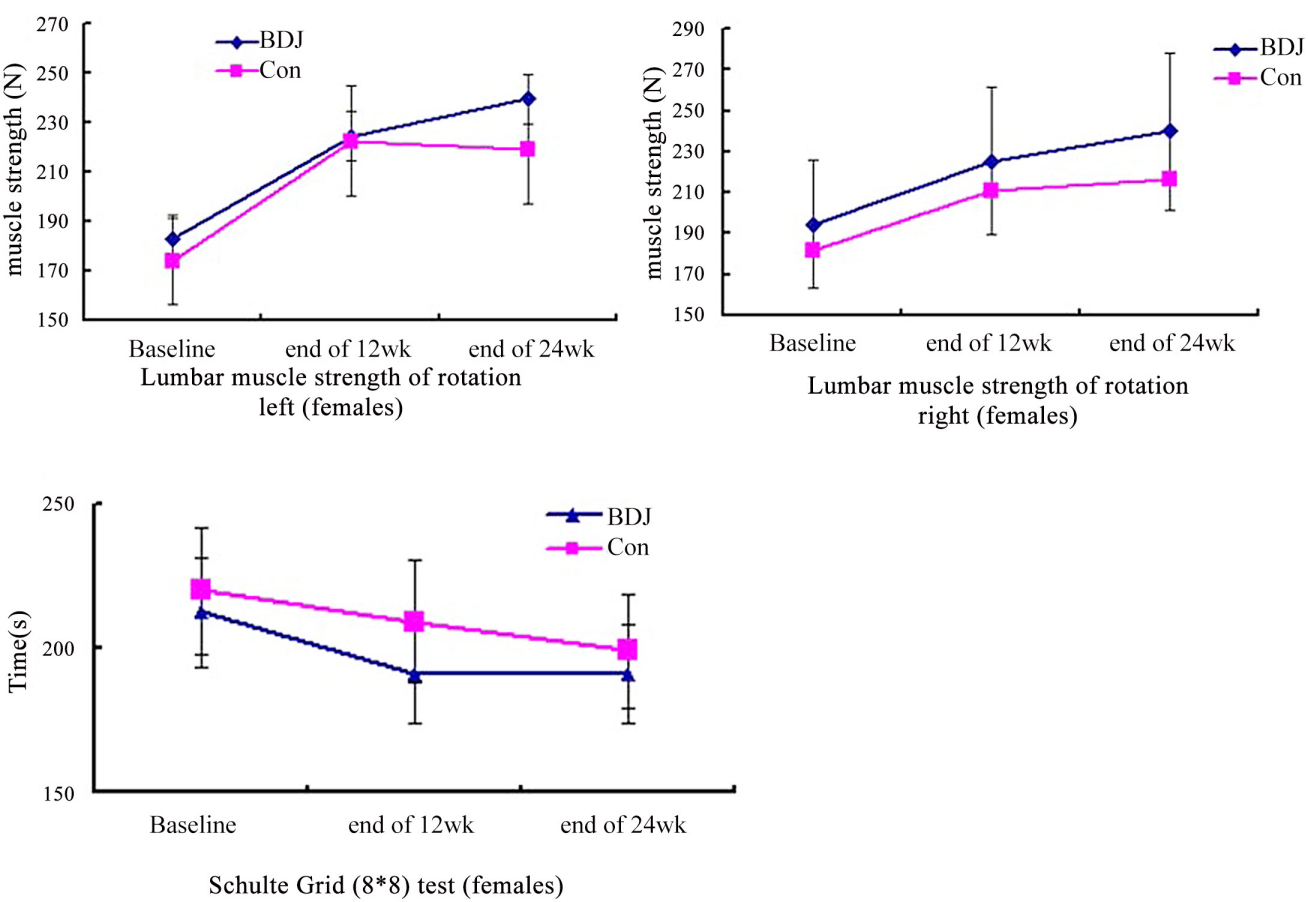
Subgroup analysis. Comparison of rotation strength of left, right lumbar muscle (Newton) in female college students between Baduanjin exercise (BDJ) and control (Con), and comparison of attention (measured by Schulte grid 8*8 test, second) in female college students between groups.

Pre-specified subgroup analysis was performed to determine whether we could identify a subgroup most likely to benefit from the Baduanjin exercise on the primary outcomes. The left and right lumbar rotation strength in female participants with Baduanjin exercise had a moderate improvement comparing to female controls at end of additional 12-week follow-up period with a mean improvement of 56.92N and 45.36N versus 44.88N and 34.83N, respectively (*P* = 0.066, and *P* = 0.042) (Fig 2). Only attention in primary well-being health outcomes in female participants of Baduanjin exercise group had a significant improvement with a mean decline 21.38 second over intervention period comparing with a mean decline 10.83 second in controls (*P* = 0.02) (Fig 2).

### 3.5 Safety

No adverse events (AEs) related to Baduanjin exercise occurred in this trial.

## Discussion

In this randomized controlled trial of Baduanjin exercise comparing with control for the physical and mental health of college students, we found significant improvement with regular Baduanjin exercise on lower limb proprioception function, cardiorespiratory endurance, flexibility, explosive force of lower limb, and attention over a 12-week intervention than usual exercises. However, significant benefits were not observed over the additional 12-week follow-up period, which indicated there was no long-term benefit to practice Baduanjin exercise. No adverse event related to Baduanjin exercise was reported during the intervention period, which suggested this form of exercise is safe.

As one of the most common forms of traditional Chinese qigong exercises, Baduanjin exercise is not only easy to learn, but also has less cognitive demanding [43]. It has been considered as a popular community exercise to promote health in China [44]. According to traditional Chinese medicine theory, good health means the unison and balance of the body and spirit. The benefits of regular Baduanjin exercise are expressed through adjusting breathing to make the process of smoother, unifying mind and breathing, strengthening muscles and tendons to make the body more flexible and the unison of mind and body [45,46]. In view of sport medicine and traditional Chinese medicine, practitioners should focus on their lumbar function, and it stresses ‘take the waist as the axis’ when practicing [47]. It is therefore expected to enhance participants’ lumbar muscle strength. In this trial, the increments of lumbar muscle strength in college students for 12 weeks of regular Baduanjin exercise are higher than those with usual exercise, however, the statistical significant difference was not observed between groups. The explanation may be that the college students in control group were already involved in high-intensity sport activities such as basketball, badminton, and ping-pong, etc. However, the enhance on lower limb proprioception function after 12 weeks of Baduanjin exercise in college students was higher than controls.

Studies in community-dwelling older population indicated that a Baduanjin exercise program could modulate blood lipid metabolism, insulin sensitivity, and sleep quality [29–31,48]. However, there are few studies focusing on its effect among young adults, especially college students. A previous study with 24 enrolled college students demonstrated an improvement on blood pressure, heart rate, lung capacity, weight, and hand grip strength after 12-week Baduanjin exercise [49]. The significant changes on vital capacity and heart rate were reported in another study with 200 college students [50]. Both studies were not randomized controlled trials, therefore it is difficult to generalize the study findings. In this randomized parallel controlled study comparing with usual exercise, the significant changes of vital capacity, blood pressure, heart rate, and hand grip force between groups were not observed. However, the effect of Baduanjin exercise on cardiorespiratory endurance, flexibility and explosive force of lower limb had obvious improvement over 12-week regular practice.

Baduanjin exercise can be described as a combination of Confucianism, Buddhism, Taoism and traditional Chinese medicine. The key point when practicing is thought to be regulation of the mind. Therefore, it requires practitioner to remove all thoughts and focus on a certain region of the body to feel the flow of Qi through the body [51]. Through the practice of Baduanjin exercise, one can expect to improve, strengthen, and gain spiritual cultivation. A previous study found that regular Baduanjin exercise with 3 times per week and 60 min per time for 8 weeks had a positive trend to improve self-image and reduce psychologic distress comparing with the inactive controls [52]. Another study from female university students suggested that Baduanjin exercise could decrease depression, fatigue, anger and tension symptoms measured by SCL-90 scale substantially, and improve self-respect, energy and emotional state after a 6-month intervention [53]. In the present trial, self-symptom intensity score in Baduanjin exercise group had a mean reduction of 7.3 points, and a mean reduction of 5.96 points in control group after 12 week exercise, but the difference was not significant between groups. Other psychological outcomes, such as stress, self-efficacy, self-esteem, mood and mindfulness, quality of life, and quality of sleep, were not different between groups either. We thought that the original activities habit practiced by the included participants, such as basketball, badminton, and ping-pong sport, might have a similar effect on psychological outcomes of college students as Baduanjin exercise. However, we did find the attention measured by the Schulte Grid test was improved with a mean shorten 7.5 second in participants over a 12 weeks of regular Baduanjin exercise comparing with controls (*P* = 0.034). This finding may be due to the fact that the exercisers were required to concentrate on adjusting breathing to make unity of mind and breathing when Baduanjin exercise was practicing.

### Study strengths and limitations

This trial focused on a unique college student population and we used a rigorous randomized, parallel-controlled design with a relatively large sample to evaluate the effectiveness and safety of Baduanjin exercise on physical and mental health. We arranged two qualified physical teachers serving as the Baduanjin exercise instructors in order to ensure the standardization of exercise training for participants. Participants in Baduanjin exercise group were gathered to do the exercise at a fixed setting and time. To control confounding factors, all participants were required to record their daily physical activity. Furthermore, the result evaluators and statistical analysts were blinded to ensure the authenticity and objectivity of the trial results. We conducted this trial according to the protocol strictly to control the quality. Therefore the internal validity of the results in this trial was achieved.

Several limitations occurred in this trial. Ideally, everyone involved in an RCT should be blinded, but this is not always feasible in the non-pharmacological trials [54]. Although the participants and exercise instructors are not blinded and the psychological outcome measures are self-report, the assessment of the related physical fitness outcomes, lumbar muscle strength and lower limb proprioception function, and the statistical analyses were performed by research staffs blinded to the treatment allocation. Second, all participants were recruited in one medical university with a greater proportion of female participants, and not controlled intensity, frequency and types of usual activities in two groups, and a relative inadequacy of adherence rate in Baduanjin exercise group. 18 participants had 75% attendance rate or less, and only 40 participants attained more than 85% attendance rate in Baduanjin exercise group. Third, the physical activity was self-report that has inherent limitations such as recall bias or social desirability. In addition, because the effect size between BDJ and Con groups was lower than anticipated, it was possible the study power was overestimated. All of which may affect the efficacy of Baduanjin intervention. Nonetheless, excellent protocol adherence, strictly performance supervision, no significant loss to follow-up and withdrawn, and the use of adjusted analyses strengthen the findings.

## Summary

In conclusion, this study demonstrates that regular Baduanjin exercise may improve lower limb proprioception function and cardiorespiratory endurance, enhance flexibility, explosive force of lower limb as well as attention in a college student population. Our finding indicates that regular Baduanjin exercise may be an effective, safe and useful form of exercise to promote the college students’ physical health and attention.

## Differences Between Protocol and Trials

1. Data of extension and lateroflexion lumbar muscle strength in all participants at baseline was lost due to accidental fault of the measurement machine (the *Tergumed Work Station*) at baseline assessment completed. They were therefore not involved in the final statistical analysis.
2. Through expert consultation, lower limb proprioception function was thought to be a better indicator to reflect the effect of Baduanjin exercise on proprioception function. As a result, lumbar proprioception function was replaced by lower limb proprioception function.
3. Standing long jump was discussed to be added in the secondary outcomes because it can excellently reflect explosive force of lower limb and also is an important component of physical fitness.

## Supporting Information

S1 CONSORT Checklist CONSORT Checklist. (DOC)

S1 Protocol Protocol of this trial (Chinese version). (DOC)

S2 Protocol Protocol of this trial (English version). (DOC)
