# Supplementary material for: The Effect of Chinese Traditional Exercise-Baduanjin on Physical and Psychological Well-Being of College Students: A Randomized Controlled Trial
Source: PLoS One. 2015 Jul 9;10(7):e0130544. doi: 10.1371/journal.pone.0130544 (PMC4497728; doi:10.1371/journal.pone.0130544)
Supplement: S1 Protocol — (DOC) [file pone.0130544.s002.doc]

**八段锦运动对大学生身体心理健康影响效果的随机对照研究计划书**

**一、研究目的**

采用随机对照设计评价八段锦对大学生身体心理健康影响的效果及安全性。

**二、研究方案与技术路线**

**1.样本量计算**

以八段锦对腰部前屈肌群肌力的改善作为主要效应指标对样本进行估算，以20名入选合格者做预实验，得出20人腰部前屈肌力的均数$\overline{X}$=258.25，σ=114.19。以肌力提高20%为预期目标，α取0.05，β取0.1，根据公式：$\begin{matrix} n_{1}=n_{2}=2\left(\frac{z_{\alpha/2}+z_{\beta}}{M_{e}-M_{c}}\right)^{2}\delta^{2} \\ \end{matrix}$得出n_1_= n_2_=101，允许10%失访率，即得出实验组、对照组各111人。

**2.研究对象招募与分组**

**2.1对象招募：**面向福建中医药大学一、二年级大学生招募合格的志愿者。

**2.2随机序列的产生、隐藏与对象分组：**根据估算的样本含量大小，由学校循证医学中心采用统计软件SAS9.1中的PLAN语句生成随机分配序列，将合格的研究对象按纳入顺序编号分为实验组和对照组。随机分配序列由课题组指定的项目管理员保管，不予其他人员知晓。

**2.3设盲与揭盲：**本研究无法对研究对象设置盲法，采用单盲法对结局评价人员、数据管理和统计分析人员设置盲法。通过SAS9.1软件设置盲底，并由项目管理员保存。待数据统计分析完成后，由项目管理员宣布组别的代码进行揭盲。

**3.纳入和排除标准**

**3.1纳入标准**

①年龄16-25岁；

②知情同意，志愿参加；

③大学一年级或二年级在读。

符合以上标准者，可纳入研究。

**3.2排除标准**

①有长期规律练习八段锦者；

②武术协会、舞蹈协会、健美操协会、散打协会、跆拳道协会成员；

③患有严重的心血管疾病、肌肉骨骼系统疾病以及其他运动禁忌症而不适宜进行八段锦运动者。

符合以上任何一条标准者,不予纳入研究。

**4.干预方法**

**4.1八段锦组:**由福建中医药大学体育部具有教练资质的体育教师带领练习八段锦。八段锦练习按照国家体育总局2003年颁布的“健身气功·八段锦”标准，动作包括两手托天理三焦、左右开弓似射雕、调理脾胃须单举、五劳七伤往后瞧、摇头摆尾去心火、两手攀足固肾腰、攒拳怒目增气力、背后七颠百病消，共8个动作。

**4.2对照组:**在自然状态下不采取任何干预措施，保持原有的生活方式。

两组在干预过程中每天均须记录当天活动情况，归类分为静态作业、低等强度活动、中等强度活动、高等强度活动。

**5.干预与随访**

**5.1干预时间：**八段锦组每次集中练习60分钟，每周练习5次，持续12周。每天练习时间为下午5点至6点。正式练习之前常规热身运动20分钟。

**5.2 随访**：随访期为12周，以参与者自我报告的形式进行随访，随访期间参与者按要求填写活动日志及相关情况表。

**6.结局指标评价**

所有结局指标在基线、干预后（第13周）、随访后（第25周）各测量一次。

**6.1主要结局指标**

①腰部肌力：包括前屈、后伸肌力，左右旋转肌力及侧屈肌力。采用德国 Proxomed公司生产的Tergumed工作站（型号：前屈工作站：Tergumed Flexion；侧屈工作站；Tergumed lateral Flexion；背伸工作站：Tergumed Extension；旋转工作站：Tergumed Rotation）测量，由福建中医药大学附属康复医院的康复治疗师进行评定。

②腰部本体功能：采用意大利 Tecnobody .S.r.l公司生产Prokin（型号PK254P）测量，由福建中医药大学附属康复医院的的康复治疗师进行评定。

③症状评分：采用SCL-90自评症状量表测量。

④压力：采用知觉压力量表（CPSS）测量。

⑤自我效能(self-efficacy)：采用一般自我效能量表测量(General Self-efficacy Scale, GSES）测量。

⑥注意力：采用舒尔特方格量表测量。

**6.2次要结局指标**

①身体素质：包括心肺功能（台阶测试、肺活量、血压、心率）、柔韧性、握力。由福建中医药大学体育部根据《大学生体质健康标准》，采用北京中体同方有限公司生产的体质测试仪进行测试。

②自尊(self-esteem)：采用自尊量表（SES）测量。

③情绪与情感（mood and mindfulness）：采用心境状态量表（POMS）测量。

④生活质量指数：采用生活质量量表（WHOQOL-BREF）测量。

⑤睡眠质量：匹茨堡睡眠质量指数（the pittsburgh sleep quality index,PSQI）

**6.3安全性指标**

记录研究过程中出现的与八段锦相关的不良事件，分析其原因，并记录处理情况。

**7.数据收集与管理**

**7.1数据收集**

研究对象的人口学资料及一般资料在招募时由对象筛选者收集，研究助理负责控制资料收集质量及数据录入。项目管理者负责将原始数据进行整理、审核、编码，并且将其转换为数据分析格式。

**7.2统计分析方法**

计量资料将采用均数±标准差或中位数（最小值，最大值）进行统计描述，t检验或秩和检验进行统计推断。计数资料采用频数（构成比）进行统计描述，x^2^检验精确概率法或非参数检验进行统计推断。亚组分析根据研究对象的性别对主要结局治疗进行。

统计分析将采用SPSS 21.0统计分析软件进行计算。所有的统计检验均采用双侧检验，p值小于或等于0.05将被认为所检验的差别有统计意义。

**【主要技术路线】**

数据统计

**干预结束，结局评价（心理健康指标、相关功能测量指标）等**

志愿者招募

根据纳入、排除标准筛选合格对象，签署知情同意书

**调查基线情况、相关功能检查、心理健康调查**

每天1次，5次/周，连续12周

对照组111例

八段锦组111例

随机化分组

随访12周，研究对象自报告结局

**随访结束，结局指标（心理健康指标、相关功能测量指标）等**

**自我报告结果**

记录不合格者人数及原因
