# Supplementary material for: The Effect of Chinese Traditional Exercise-Baduanjin on Physical and Psychological Well-Being of College Students: A Randomized Controlled Trial
Source: PLoS One. 2015 Jul 9;10(7):e0130544. doi: 10.1371/journal.pone.0130544 (PMC4497728; doi:10.1371/journal.pone.0130544)
Supplement: S2 Protocol — (DOC) [file pone.0130544.s003.doc]

**The effect of Baduanjin exercise on physical and psychological wellbeing of college students: study protocol for a randomized controlled trial**

1. Study object

The primary aim of this trial is to evaluate the effectiveness and safety of Baduanjin exercise on physical and mental health of college students through a rigorous randomization, parallel-controlled design.

2. Method/design

2.1 Sample size

We use the improvement of lumbar flexion muscle strength as the main effect indicators to estimate the sample size, and our preliminary test data from 20 eligible participants indicated the means with standard deviation of the lumbar flexion muscle strength was 258.25 newton (N) and 114.19 N respectively. To detect a 20% mean difference on the Baduanjin exercise at the end of intervention, 101 participants per group are calculated according to the formula $\begin{matrix} n_{1}=n_{2}=2\left(\frac{z_{\alpha/2}+z_{\beta}}{M_{e}-M_{c}}\right)^{2}\delta^{2} \\ \end{matrix}$ with a type I error of 5% (α= 0.05) and 90% power (β= 0.10). Assuming a dropout rate of 10% for follow-up, the sample size is 111 for each group.

2.2 Participant and recruitment

We will recruit 222 college students from Fujian University of Traditional Chinese Medicine (FJTCM).

2.2.1 Inclusion criteria

(1) be aged 18 to 25 years;

(2) be able to give the informed consent form;

(3) be a fulltime student at first or second grade.

2.2.2 Exclusion criteria

(1) being or having been engaged in a long-term regular practice of Baduanjin;

(2) being a member of the Martial Arts Association, Dance Association, Aerobics Association, Sanda Association, or Taekwondo Association;

(3) those who have suffered from severe cardiovascular diseases, musculoskeletal system diseases, or other sports contraindications.

2.3 Randomization, allocation concealment and blinding

The random allocation sequence will be produced by an independent statistician using the statistical software SAS9.1. The participants will be allocated to either Baduanjin exercise or usual exercise control group according to 1:1 equal proportion rule. The random allocation sequence will be managed by a specified project manager who is not involved in the recruitment program.

The random allocation sequence will be managed by the project manager. The project manager and exercise coaches will be not involved in the assessment of outcome; and the outcome assessors and the statistic analyzer will be not involved in the participants’ screening and allocating.

2.4 Intervention

2.4.1 Baduanjin exercise group

A 12-week Baduanjin exercise training (1 h per day from 5 p.m. to 6 p.m., 5 days per week) will be applied to the participants in the Baduanjin exercise group. Two qualified coaches who have engaged in the physical education over 5 years will teach the participants the correct Baduanjin postures according to the *Health Qigong - Baduanjin*, published by the General Administration of Sport of China. The ten postures are *Preparation posture, Prop up the sky with hands to regulate the triple* *energizer, Draw a bow on both sides like shooting a vulture, Raise single arm to regulate the Spleen (Pi) and Stomach (Wei), Look back to treat five strains and seven impairments, Sway head and buttocks to expel Heart (Xin)-fire, Pull toes with both hands to reinforce the Kidney (Shen) and waist, Clench fists and look with eyes wide open to enhance strength and stamina, Rise and fall on tiptoes to dispel all diseases*, and *Ending posture*.

2.4.2 Control group

The participants in the control group will not receive any specific training. They will keep their original exercises.

All participants will be required to record their daily activity or sport information during the intervention period, which is classified into low, moderate, or high intensity grade.

2.5 Follow-up

All participants will keep their usual exercises and record their daily activity or exercise information.

2.6 Outcome assessment

All primary and secondary outcomes will be assessed at baseline, 13 weeks (end of intervention), and 25 weeks (after the 12-week follow-up period).

2.6.1 Primary outcomes

• Lumbar muscle strength consists of flexion, extension, lateroflexion, and rotation myodynamia. It will be assessed with the *Tergumed Work Station* (produced by Proxomed GmbH, Germany; product type: Flexion Work Station:Tergumed Flexion; Lateral Flexion Work Station: Tergumed lateral Flexion; Extension Work Station: Tergumed Extension; Rotation Work Station: Tergumed Rotation) by experienced operators at the Evaluation Department of Rehabilitation Hospital Affiliated to FJTCM.

• Lumbar proprioception function will be measured by the *Prokin* proprioception evaluation and training system (produced by Tecnobody .S.r.l, Italy; product type: PK254P) by the Evaluation Department of Rehabilitation Hospital Affiliated to FJTCM.

• The Symptom Checklist-90 (SCL-90) will be used to measure self-reported symptom intensity.

• Stress will be measured with the Chinese Perceived Stress Scale (CPSS).

• Self-efficacy will be assessed with General Self-efficacy Scale (GSES).

• Attention will be assessed by Schulte Grid (8*8) test.

2.6.2 Secondary outcomes

• Physical fitness consists of cardiopulmonary function (including the step test, vital capacity, blood pressure, and heart rate), flexibility, and hand grip strength. It will be performed by the Department of Physical Education in FJTCM with physical fitness tester produced by Zhongtitongfang Co., Ltd., Beijing, according to the Chinese University Students’ Physical Health Standard.

• Mood and mindfulness will be measured by Profile of Mood States (POMS).

• The Self-Esteem Scale (SES) will be administered to measure self-esteem.

• Quality of life will be measured by the World Health Organization Quality of Life - BREF (WHOQOL-BREF).

• Quality of sleep will be measured by the Pittsburgh Sleep Quality Index (PSQI).

2.6.3 Safety measurements

Any adverse events related to Baduanjin, and how they are addressed, will be recorded during the intervention period.

2.7 Data collection and management

The demographic and baseline characteristic data will be collected by screeners when the participants are recruited. Research assistants will conduct quality control of data collection and be responsible for data entry. The project manager will be responsible for initial data cleaning, identifying, coding, and converting into the proper format for data analysis.

2.8 Statistical analysis

Baseline characteristics and outcomes between groups will compare using the t-test or non-parametric test for continuous variables and Pearson chi-squared or Fisher’s exact test for categorical variables. Subgroup analysis stratified by participants’ sex will be used for the primary outcomes.

All data will be analyzed with SPSS 21.0 (IBM, Chicago, IL, USA) software packages. The statistical significance is defined as two-sided P value of <0.05.

Participants recruitment

Eligible participants n=222

Baseline assessment

Exclusion (reasons): n=

Not meeting inclusion criteria: n=

Declined to participate: n=

Other reasons: n=

Randomization allocation

Baduanjin training group

n=111

Control group

n=111

Drop out (reasons): n=

Intervention group: n=

Control group: n=

Outcomes assessment after intervention: lumbar muscle strength, lumbar proprioception function, self-reported symptom intensity, stress, self-efficacy, attention, cardiopulmonary function, flexibility, hand grip strength, self-esteem, mood and mindfulness, quality of life, quality of sleep and adverse events

Follow up for 12 weeks

Lost to follow up: n=

Intervention group: n=

Control group: n=

Above outcomes assessment after follow-up period

Included analysis: n=

Intervention group: n=

Control group: n=

Statistic analysis

Excluded from analysis: n=

Intervention group: n=

Control group: n=

**Figure 1 Flow diagram of participants**
